# Supplementary figures and images for: Single-cell RNA-seq reveals CD16- monocytes as key regulators of human monocyte transcriptional response to Toxoplasma
Source: Sci Rep. 2020 Dec 3;10:21047. doi: 10.1038/s41598-020-78250-0 (PMC7713135; doi:10.1038/s41598-020-78250-0)

Figure S1

**A**

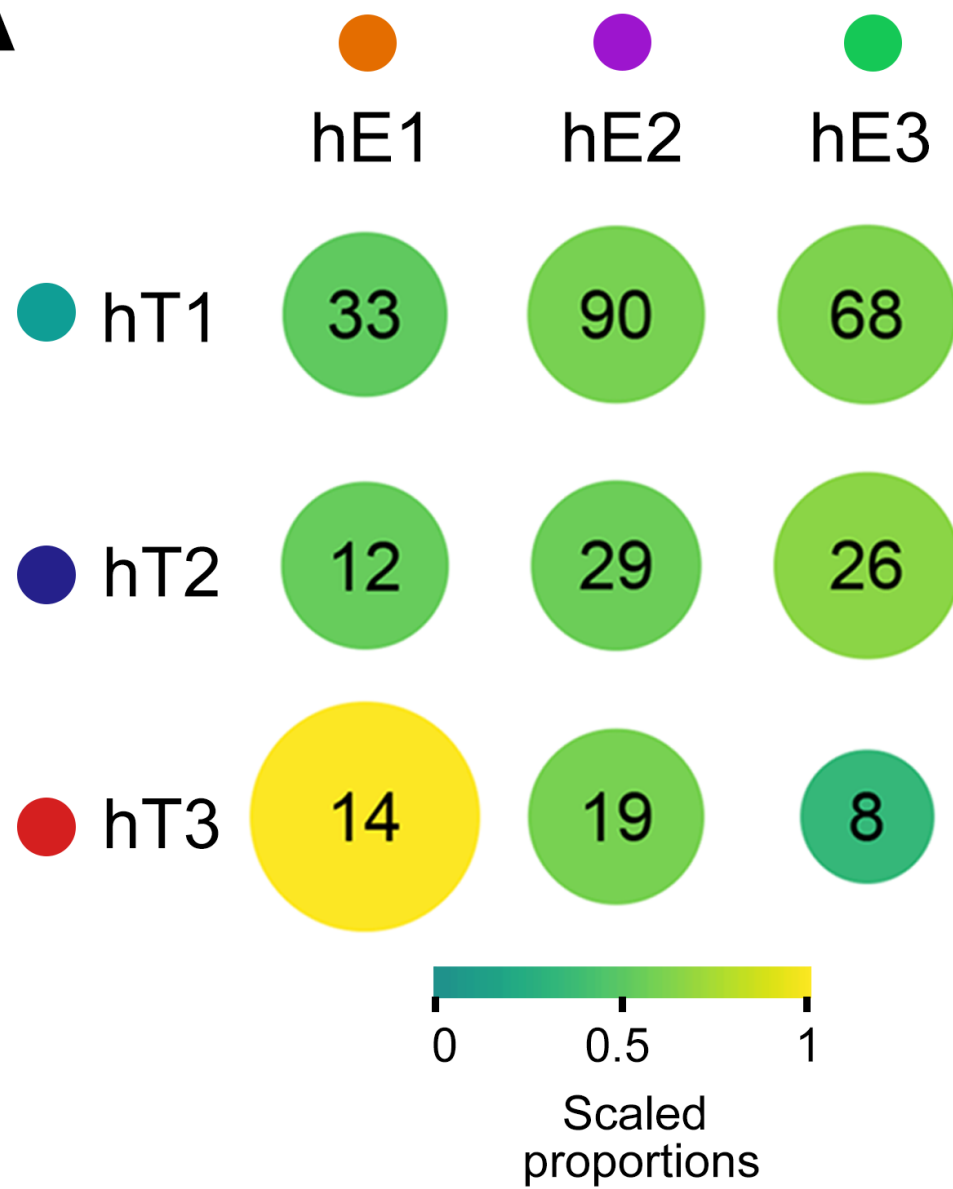

**B**

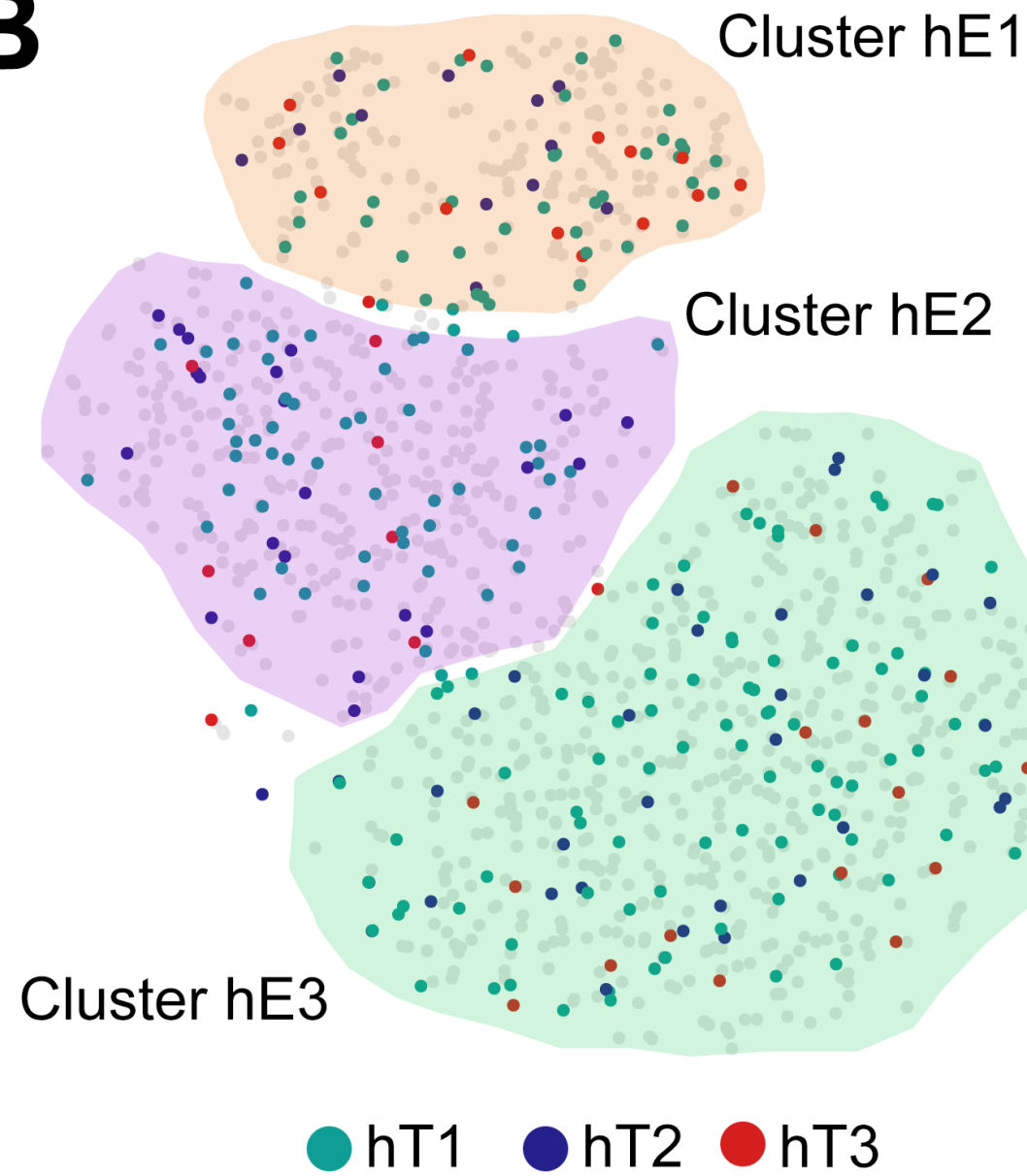

Supplement: Supplementary file 1 — Supplementary Information 1. [file 41598_2020_78250_MOESM1_ESM.pdf]
