# Supplementary material for: Single-cell RNA-seq reveals CD16- monocytes as key regulators of human monocyte transcriptional response to Toxoplasma
Source: Sci Rep. 2020 Dec 3;10:21047. doi: 10.1038/s41598-020-78250-0 (PMC7713135; doi:10.1038/s41598-020-78250-0)
Supplement: Supplementary file 4 — Supplementary Information 4. [file 41598_2020_78250_MOESM4_ESM.docx]

## S1: Differential expression and enrichment analysis for monocyte subsets: A) Differentially expressed genes in monocytes groups (infected or uninfected) within each cluster. B) GO terms (biological processes & cellular localization) enriched in genes DE expressed in infected or uninfected cells within each monocyte cluster

## S2 Differentially expression and enrichment analysis for *Toxoplasma* clusters: A) Differentially expressed genes in individual *Toxoplasma* clusters. B) GO terms enriched in the DEGs.
